# Impaired Global, and Compensatory Local, Biological Motion Processing in People with High Levels of Autistic Traits

**DOI:** 10.3389/fpsyg.2013.00209

**Published:** 2013-04-23

**Authors:** Jeroen J. A. van Boxtel, Hongjing Lu

**Affiliations:** ^1^Psychology Department, University of California Los AngelesLos Angeles, CA, USA; ^2^Statistics Department, University of California Los AngelesLos Angeles, CA, USA

**Keywords:** biological motion perception, autism spectrum disorder, attention, individual differences, predictive coding, adaptation, dual-task

## Abstract

People with Autism Spectrum Disorder (ASD) are hypothesized to have poor high-level processing but superior low-level processing, causing impaired social recognition, and a focus on non-social stimulus contingencies. Biological motion perception provides an ideal domain to investigate exactly how ASD modulates the interaction between low and high-level processing, because it involves multiple processing stages, and carries many important social cues. We investigated individual differences among typically developing observers in biological motion processing, and whether such individual differences associate with the number of autistic traits. In Experiment 1, we found that individuals with fewer autistic traits were automatically and involuntarily attracted to global biological motion information, whereas individuals with more autistic traits did not show this pre-attentional distraction. We employed an action adaptation paradigm in the second study to show that individuals with more autistic traits were able to compensate for deficits in global processing with an increased involvement in local processing. Our findings can be interpreted within a predictive coding framework, which characterizes the functional relationship between local and global processing stages, and explains how these stages contribute to the perceptual difficulties associated with ASD.

## Introduction

What it means to “see” has been succinctly characterized as “to know what is where by looking” (Marr, 1982). The visual system is constantly inundated by a vast array of cues containing information at different levels of analysis. In a soccer game, for example, when your opponent is about to pass a soccer ball to a teammate, you need to rapidly predict the direction of the pass and take appropriate actions. Different types of visual cues can be used for this task. You could use local cues, focusing just on the movement of the most important joint (e.g., the ankle). Alternatively, you could use global cues, such as the body posture (e.g., whether the opponent’s body is directed leftward or rightward) and overall body movements. To extract and analyze different cues such as these, the visual system consists of several processing stages, often broadly divided into low-level and high-level stages. The low-level stages perform local, detail-oriented processing of relatively simple stimulus features (e.g., orientation of body limbs or motion trajectory of individual joints), while high-level stages perform more global, contextual, and configural processing of stimuli (e.g., perceiving body structure and movements, action recognition, or interpreting social cues).

Relatively little research has been directed at individual differences in how different stages may contribute to perception for typically developing observers, even though studies with clinical populations demonstrate the importance of individual differences, at the extreme end of the spectrum. In particular, the distinct contributions of local versus global stages have been highlighted in work on autism spectrum disorder (ASD) (Plaisted, 2001; Happé and Frith, 2006; Mottron et al., 2006). To explain abnormalities in the extraction of meaningful social cues from visual stimuli, it has been proposed that people with ASD have superior low-level processing but a relative deficit in high-level processing (Happé and Frith, 2006; Mottron et al., 2006), causing them to focus on non-social stimulus contingencies while missing important social cues (Klin et al., 2009). Although people with autism may have trouble in social contexts, and with certain contextual perceptual tasks, the detail-oriented nature of people with ASD confers a real advantage in certain tasks that require contextual information to be ignored. For example, it is generally reported that observers with ASD are better at finding embedded figures (i.e., a local figure that is part of a global drawing) than neurotypics (Shah and Frith, 1983; Simmons et al., 2009), suggesting that they are less distracted by the global drawing. This task, and related visual search tasks, have also showed that within the typical population, people with more autistic traits [as measured with the autism spectrum quotient, AQ (Baron-Cohen et al., 2001)], are better and faster than people with fewer autistic traits (Grinter et al., 2009a,b; Almeida et al., 2010a,b; Russell-Smith et al., 2012). However, it should be noted that the overall literature is still equivocal for the embedded figures task (White and Saldana, 2011). Similarly, there is mixed evidence from face processing studies (e.g., identity, emotion) in ASD (Pellicano et al., 2007; Simmons et al., 2009; Harms et al., 2010).

Findings concerning other perceptual characteristics in ASD, especially those related to processing of dynamic information, are also heterogeneous, but to a lesser degree (Dakin and Frith, 2005; Pellicano et al., 2005; Kaiser and Shiffrar, 2009; Simmons et al., 2009). People with ASD generally perform normally on simple luminance-defined motion tasks, but they have been reported to perform worse for second-order (contrast-defined) motion detection tasks (Bertone et al., 2003). Inconsistent findings have been reported for studies that have investigated global motion perception by measuring motion coherence thresholds (Spencer et al., 2000; Milne et al., 2002; Pellicano et al., 2005; Del Viva et al., 2006), which may be attributable to stimulus and methodological differences (discussed in Simmons et al., 2009). However, the overall balance of evidence suggests that people with ASD show impairment in some forms of dynamic processing (Kaiser and Shiffrar, 2009).

Among studies using biological motion stimuli (which are rich in social cues; Dittrich et al., 1996; Chouchourelou et al., 2006; Roether et al., 2009; Manera et al., 2010; O’Toole et al., 2011), it was initially found that perception was not impaired in ASD, but that people with ASD were less likely than controls to describe the emotional contents of a stimulus (Moore et al., 1997). Later studies mostly reported that biological motion perception was impaired in ASD (e.g., Blake et al., 2003; Klin et al., 2009; Annaz et al., 2010; Nackaerts et al., 2012), in addition to having reported problems in describing/discriminating emotions (Hubert et al., 2007; Parron et al., 2008; Nackaerts et al., 2012). Nevertheless, there are also some reports that do not find differences between ASD and typical populations (Murphy et al., 2009; Saygin et al., 2010). It is noteworthy that many of the studies that did not find significant differences used older populations, suggesting that age may be an important covariate in determining the behavioral effects of ASD (Kaiser and Pelphrey, 2012), in the sense that older observers with ASD may have developed some compensatory mechanism to overcome deficits and therefore show the same level of behavioral performance as the typical population. However even without overt differences in behavior, several imaging studies report significant differences in brain activity (Herrington et al., 2007; Freitag et al., 2008; Kaiser et al., 2010; McKay et al., 2012). Most interestingly, the brain regions showing activity difference between ASD and typical populations include the posterior superior temporal sulcus (pSTS), one of the main regions in the brain underlying social perception and social cognition (Adolphs, 1999; Puce and Perrett, 2003), and it is this area (STSp) that is increasingly sensitive in the perception of human movements for typical children (Carter and Pelphrey, 2006), but not in young observers with ASD (Pelphrey and Carter, 2008).

Why do the two populations often show similar behavioral performance in psychophysical experiments, yet different brain activities in key regions of biological motion perception? A recurring problem is that, in most biological motion tasks (as well as tasks related to face recognition), normal performance can be achieved by using either local or global processes that utilize different stimulus characteristics. A deficit in one process may therefore be masked by increased involvement of the other process. Such compensation could provide a potential reason why older observers with ASD show smaller behavioral effects: older observers may have learned to use superior processing with local cues to compensate for the absence of or decrease in global processing. Consequently, it is often unclear whether high performance indicates that the observer has intact global processing, or has deficient global processing that is compensated for by an increase in local processing. Such ambiguity contributes to controversy regarding how people with ASD differ from typical observers in their visual analyses (Dakin and Frith, 2005; Pellicano et al., 2005; Kaiser and Shiffrar, 2009; Simmons et al., 2009). These same issues are at play in the typical population, where large individual differences likely exist as well.

Two important issues yet to be resolved are: (1) what is the nature of the deficit in global processing? Is it a “hard-wired” deficit in global processing, or a more cognitive bias favoring local processing? (2) What are the contributions of local and global perceptual processes to the perceptual and social problems of those with ASD and in the typical population? In the present study we conducted a detailed psychophysical investigation of the contributions of local and global processing to biological motion perception. Biological motion has several important characteristics that make it exquisitely suitable for this investigation. First, it is known to involve both local and global processing levels, which allows us to use a single type of stimulus to investigate both levels (Hirai and Kakigi, 2008; Chang and Troje, 2009). For example, local processing of the movement of a single joint is sufficient for observers to discriminate between left and rightward walkers (Troje and Westhoff, 2006). Global processing is revealed by the significant influence of configural information on biological motion perception (Pavlova and Sokolov, 2000; McKay et al., 2009). Second, biological motion is one of the main sources of social cues (Poizner et al., 1981; Dittrich et al., 1996; Chouchourelou et al., 2006; Roether et al., 2009; Manera et al., 2010), which increases the likelihood of finding a correlation with the number of autism-like traits. Third, previous studies established the possibility of examining differences between typical individuals with fewer and more autistic traits in biological motion perception. Studies have reported that people with many autistics traits are less sensitive to biological motion stimuli (Kaiser and Shiffrar, 2012) and show higher motion thresholds (Grinter et al., 2009a). In our studies, we correlated observed perceptual measures with the number of autistic traits displayed by healthy adult observers (as measured by a 50-point AQ score; Baron-Cohen et al., 2001), allowing us to determine the impact of autistic traits on observers’ abilities to process biological motion information at multiple levels.

## Experiment 1: Impairment of Automatic Global Processing is Associated with Increased Number of ASD Traits

**Figure 1:**
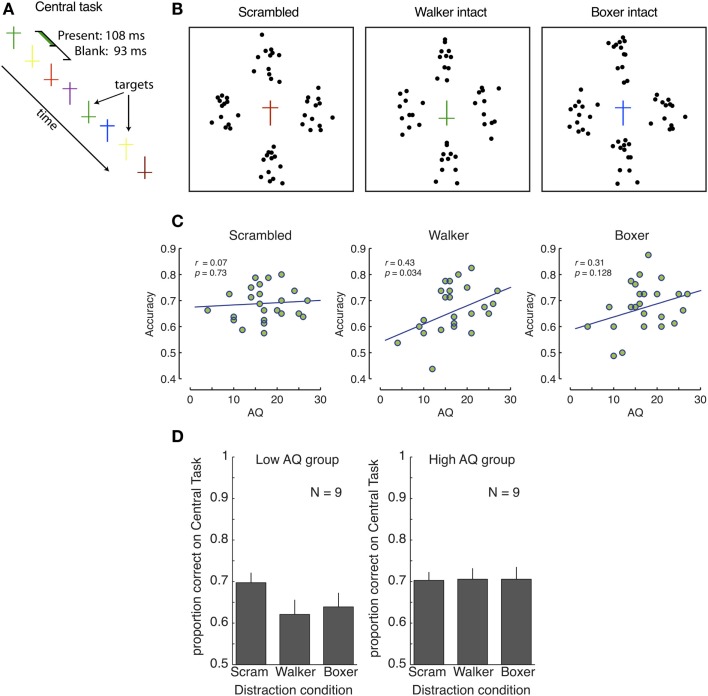
**No involuntary, automatic, direction of attention to global biological information**. **(A,B)** Experimental design. **(A)** The central task consisted of counting the upright yellow and inverted green crosses; there were always one or two targets present. **(B)** The central task was surrounded by either four scrambled actions (two scrambled walkers, and two boxers), or two intact walkers or boxers (supplemented by two scrambled actions of the other action type). **(C)** Correlations between AQ and accuracy in the central task for scrambled (left), walker (middle), and boxer (right) actions. **(D)** Central task performance for low and high-AQ groups. Performance in the scrambled condition was comparable between groups, demonstrating no advantage for the potentially detail-oriented high-AQ group. With intact biological motion displays, the performance decreased significantly for the low-AQ group. No such decrease occurred for the high-AQ group, indicating that they were not distracted by, and did not process, the global aspects of the peripheral biological motion stimuli. Error bars represent SEM.

In Experiment 1, we investigated if, in a typical population, global biological motion information automatically attracts attention, and how this differs among individuals. Typical healthy adults are reported to be automatically (pre-attentively) attracted to biological motion stimuli (Thornton and Vuong, 2004), even when it is detrimental to the task at hand, suggesting a “hard-wired” need to process biological motion. We hypothesized that this type of incidental processing varies among individuals, and is negatively related to the degree of autism. We tested this hypothesis by asking observers to perform a central counting task at fixation (see Figure 1A; Bahrami et al., 2008; van Boxtel et al., 2010), while task-irrelevant biological motion stimuli (point-light walker or boxers) were present in the periphery (see Figure 1B). The behavioral measure (accuracy on the central task) quantifies the interference of task-irrelevant biological motion stimuli on the center task performance. Importantly, we contrasted conditions in which the peripheral point-light stimuli were either intact or spatially scrambled. This critical manipulation ensures that local motion information (i.e., trajectories of joint movements over time) was identical, and only global information (e.g., body structure) about actions varied among conditions. As a result, any observed effect of the task-irrelevant stimuli can be attributed to differences in global processing. We expected that performance of the central task would deteriorate in the presence of task-irrelevant intact biological motion stimuli, but more so for individuals with low levels of autistic traits. We also hypothesized that the boxing action would be more distracting (thus resulting in lower accuracy on the central task) than the walking action, based on our previous finding that boxers are more easily detected in visual search displays than walkers, and therefore probably possess an attention-grabbing feature (van Boxtel and Lu, 2011, 2012).

### Methods

#### Subjects

Twenty-five observers [21 female; mean (std) ages = 19.6 (1.2), based on 11 observers whose age data were collected] gave informed consent. Observers were UCLA psychology undergraduate students, who participated for course credit. Participants filled out a questionnaire from which we derived an AQ (Baron-Cohen et al., 2001). People who have many characteristics consistent with ASD will have a high-AQ score, whereas those who have few such characteristics will have a low score. AQ scores ranged from 4 to 27 (mean = 16.88, std = 5.54, median = 17). We did not ask whether participants were diagnosed with ASD.

#### Stimuli

Point-light stimuli were generated from a free online motion-capture database (http://mocap.cs.cmu.edu) (van Boxtel and Lu, 2011). We employed walker and boxer actions in Experiment 1 (3.5° in height). Stimuli were displayed at 3.5° or 7° eccentricity. There were no significant influences of eccentricity, so the data were collapsed over this variable. We displayed two walkers (either above/below fixation, or left/right of fixation) and two boxers (displayed at the remaining two position, above/below or left/right of fixation). In intact conditions, one of the two action types was intact, the other was scrambled; in scrambled conditions, all four stimuli were scrambled. This manipulation ensured that local motion information was identical in all experimental conditions, and only global information differed.

#### Procedure

Subjects performed an engaging central task during the stimulus presentation. They were asked to count upright yellow and inverted green crosses (1° wide, 1.5° high, line thickness: 0.18°, horizontal bar offset from center: 0.36°), among upright and inverted crosses of eight different colors (van Boxtel et al., 2010). Crosses were shown for 108 ms (eight frames), and separated by 93 ms blanks. There were one or two target crosses present, separated by at least one intervening cross. The trial lasted for 1 s, after which the subject reported the number of counted crosses by pressing “1” or “2” on the keypad. The next trial was started automatically after a delay of 500 ms. A total of 240 trials were run per observer [3 distractor types (scrambled, intact walker, intact boxer) × 2 amounts of target crosses (1 or 2) × 2 peripheral locations (3.5° or 7° eccentricity) × 20 repetitions].

### Results

A one-way ANCOVA on distractor type (Scrambled, Walker, Boxer) with AQ score as a covariate revealed a significant main effect of distractor type [*F*(2, 46) = 5.525, *p* = 0.007], and a significant effect of AQ score [*F*(2, 46) = 4.076, *p* = 0.023]. Correlation analyses confirmed that in both conditions with global biological motion information, the performance on the central task increased with AQ score [*r* = 0.427, *p* = 0.034 (walking), and *r* = 0.313, *p* = 0.128 (boxing)], while there was no such correlation for the scrambled stimuli (*r* = 0.07, *p* = 0.726), see Figure 1C. We also looked at correlations between accuracy and the five sub-factors of the AQ scores (see, e.g., Russell-Smith et al., 2012), but no correlation was significant after correction for multiple comparisons.

In order to obtain a clearer understanding of the differences among observers with few and many autistic traits, we divided the observer pool into two equally sized groups [one with low and with high-AQ scores, using the nine subjects with the lowest (≤15) and highest (≥18) AQ scores^1^; see Figure 1D]. We then performed a mixed-design repeated-measures ANOVA analysis. This ANOVA revealed a significant effect of distractor type [*F*(2, 32) = 4.036, *p* = 0.027, $\eta_{p}^{2}=0.201$], and a significant interaction with AQ group [*F*(2, 32) = 4.720, *p* = 0.016, $\eta_{p}^{2}=0.228$]. As shown in Figure 1D, for the low-AQ group with fewer autistic traits, performance in the scrambled condition was significantly higher than both the walker condition [*t*(8) = 4.063, *p* = 0.004, Cohen’s *d* = 0.84] and boxer condition [*t*(8) = 3.585, *p* = 0.007, Cohen’s *d* = 0.66], demonstrating strong interference between the central task and biological motion perception for observers with fewer autistic traits. Contrary to our expectation, the distracting effect was not greater for boxing action than for walking actions [*t*(8) = −0.893, *p* = 0.40, Cohen’s *d* = −0.17]. In fact, individual performances in both conditions are strongly correlated (*r* = 0.8, *p* < 0.0001, the line *x* = *y* has an *R*^2^ = 0.59). We conjecture that, although boxing stimulus includes critical features which attract attention automatically to interfere with the central task performance, the familiarity to the walker stimuli may also trigger incidental processing so that less attention is allocated to the central task. These results indicate that individuals with few autistic traits process global information of biological motion even when the stimuli are task-irrelevant, consistent with previous findings (Thornton and Vuong, 2004). In contrast, observers with more autistic traits (high-AQ group) failed to show such interference effects, as indicated by the identical performance on the central task in the scrambled and the two intact conditions [*t*(8) = −0.151, *p* = 0.884, and *t*(8) = −0.117, *p* = 0.910, respectively]. Thus observers with high levels of autistic traits were not distracted by the biological motion stimuli. The discrepancy between the low and the high-AQ groups suggests that, within the typical population, people with high levels of autistic traits have diminished involuntary global biological motion processing as compared to people with low levels of such traits.

In our population of Psychology undergraduate students in Experiment 1, 4 out of 25 participants were males, who generally have higher AQ scores. A few males with high-AQ scores could potentially cause our pattern of results, which would then not reflect an influence of AQ *per se*, but a gender difference. Even though in Experiment 1, there was an AQ difference between males and females [22.0 (*n* = 4) vs. 15.9 (*n* = 21); *t*(23) = −2.17, *p* = 0.04], so we analyzed our data excluding all male participants; the pattern of results (i.e., whether significance was reached for the effects) did not change.

## Experiment 2: Decreased Global Processing can be Offset by Compensatory Local Processing

Experiment 1 was designed to specifically gage whether the level of autistic traits influences the involvement of (automatic) global processing in biological motion perception. In other situations, however, decreased global processing may be offset by an increase in local processing, potentially masking any deficits in biological motion perception. In Experiment 2 we aimed to (1) replicate the findings of Experiment 1 using a different experimental paradigm, and (2) identify any potential compensatory local processing employed by individuals with high levels of autistic traits (high-AQ group).

Many previous studies have demonstrated that biological motion perception is dependent on configural, gestalt-like processes at the global processing level. For example, observers can easily identify and adapt to gender information contained in biological motion point-light walkers (Jordan et al., 2006; Troje et al., 2006), even though local information (i.e., movements of individual dots) does not contain the gender information. A key characteristic apparent in behavioral tests that distinguishes global aftereffects originating from high-level processes from conventional aftereffects in visual perception is the space-invariance of adaptation. Because lower levels in the visual system process location-specific information, locally adapted neurons are not involved in the perception of the stimulus that is presented at another location. Accordingly, adaptation effects originating at low levels are only observed when adapting and testing locations are the same. By contrast, global aftereffects induced by changes in high-level visual processes can transfer across different retinal locations (Webster, 2011). In studies that employ adaptation paradigms to test for transfer of aftereffects to different locations, it has been found that complex stimuli show a larger invariance to changes in retinal location, as shown for faces (Leopold et al., 2001; Melcher, 2005; Kovacs et al., 2008), global motion (Snowden and Milne, 1997; Melcher and Morrone, 2003), and biological motion (Theusner et al., 2011). Therefore, testing for location transfer of adaptation effects makes it possible to tease apart the involvement of global and local processing in biological motion perception. To specifically quantify the involvement of global and local visual processes, we therefore measured location-invariant and location-specific adaptation, respectively.

### Methods

#### Subjects

Thirty observers [26 female, mean (std) age 19.6 (1.5) years] participated in Experiment 2, 10 of the participants also participated in Experiment 1. Participants were undergraduate students at the UCLA psychology department and received course credit for their participation. AQ scores ranged from 9 to 27 (mean = 17.9, std = 4.66, median = 18). We did not ask whether participants were diagnosed with ASD.

#### Stimuli and procedure

**Figure 2:**
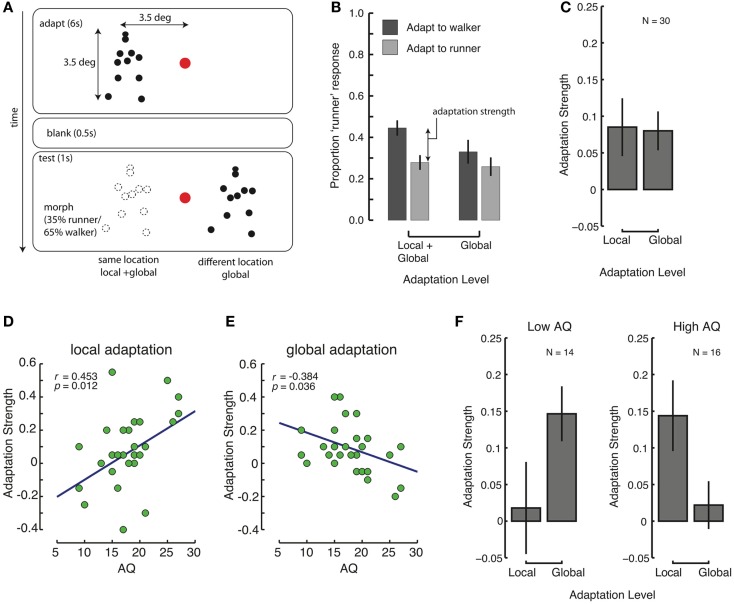
**Reduced global, and increase local processing with high levels of autistic traits**. **(A)** Design of Experiment 2. Subjects adapted to a walker or a runner, left or right of the fixation mark. After 6 s, a 500 ms blank, and then a test stimulus followed. The test could appear at the same-location as the adaptor (“same”) or at the opposite site of the fixation mark (“different”). **(B)** Average proportion of “runner” responses after adaptation to walkers (dark bars) and runners (light bars), in conditions in which the adaptation and test location were identical, and different. **(C)** Overall strength of local and global adaptation. **(D,E)** Correlations between AQ and local **(D)** and global **(E)** adaptation strengths. **(F)** Contributions of local and global adaptation for people with few (left panel) and many (right panel) autistic traits. Error bars represent SEM.

Walker and runner point-light animations (dot size = 0.27° diameter) were displayed from a sagittal view at 3.5° eccentricity either left or right of the fixation mark (red, 0.3° diameter). Each trial started with a 6-s adaptation to either a walker or runner action, displayed in black on an intermediate gray background (see Figure 2A). Gait cycle length was equalized between walker and runner actions (1 s). After a 500-ms blank, a 1-s test stimulus was displayed in white. Subjects indicated whether the test stimulus was a walker or a runner. The test was a morph between a walker (weight 0.65) and a runner (weight 0.35), calculated according to a previously described morphing algorithm (Giese and Poggio, 2000), starting at a random position in the gait cycle. We chose a weight unequal to 0.5 so that we could distinguish random responses (which would yield 50% running responses) from no-adaptation responses (which would yield 35% running responses). Adaptation and test occurred on the left and right sides of fixation independently. We collapsed the data over left and right variations, resulting in conditions in which adapt/test locations were the same (left/left, or right/right), or different (left/right, or right/left). The actions moved rightward for 13 subjects, and leftward for 17 subjects. A mixed-design repeated-measures ANOVA revealed no significant influence of movement direction, nor any significant interactions, and the data were therefore combined. A total of 80 trials were run per observer [2 adaptor types (walking, running) × 2 adaptation positions (left, right of fixation) × 2 test locations (left, right of fixation) × 10 repetitions].

### Results

The average proportion of “runner” responses across all conditions was close to 35%, indicating that the observers performed that task as instructed, rather than simply guessing. As shown in Figure 2B, after adapting to a walker (runner), the perception of a morphed walker/runner was biased toward a runner (walker) relative to the mean response (35%). The strength of this adaptation was quantified as the difference of response proportions between the two adapting conditions (Figure 2B). We assume that space-invariant adaptation is generally due to adaptation at high levels within the visual hierarchy that process global information (Kovacs et al., 2008). Hence, the strength of global level adaptation is indicated by the adaptation effect measured when the adaptation and test locations were different. In contrast, adaptation effects for the same adaptation and test locations resulted from *both* low-level processes (i.e., local adaptation) and high-level processes (i.e., global adaptation). Accordingly, in order to quantify local adaptation effects, we calculated local adaptation strength by subtracting the measures in the different-location condition (global) from those in the same-location condition (global + local). We found that both local and global adaptation were evident, with significantly above-zero adaptation strengths [Figure 2C; local: *t*(29) = 2.19, *p* = 0.038, Cohen’s *d* = 0.40; global: *t*(29) = 3.06, *p* = 0.005, Cohen’s *d* = 0.56].

A one-way ANCOVA on adaptation level (local/global) with AQ score as a covariate revealed a significant main effect [*F*(1, 28) = 8.947, *p* = 0.006], and a significant effect of AQ score [*F*(1, 28) = 9.699, *p* = 0.004]. Because of the prominent influence of AQ scores, we looked at the dependence of local and global adaptation on AQ scores using regression analyses. We found that global adaptation was significantly negatively correlated with AQ scores (*r* = −0.384, *p* = 0.036, Figure 2E), whereas local adaptation was significantly positively correlated with AQ scores (*r* = 0.453, *p* = 0.012, Figure 2D). In fact, there was a significant inverse correlation between local and global level effects (*r* = −0.382, *p* = 0.037), indicating that those observers who showed weak global adaptation showed an increased amount of local adaptation. We correlated the sub-factors of the AQ score with the local and global adaptation effects. None of the correlations survived Bonferroni corrections, but the correlation between the “social skills” measure and the local adaptation effect was at trend level after correction (*r* = 0.498, *p* = 0.051).

As in Experiment 1, we split the observer group based on AQ scores (using a median (AQ = 18) split) into low- and high-AQ groups (observers with AQ = 18 were included in the high-AQ group), and we completed a mixed-design repeated-measures ANOVA analysis (see Figure 2F). There was no overall significant effect of adaptation level (local/global) [*F*(1, 28) < 1], but there was a strongly significant interaction with AQ group [*F*(1, 28) = 6.220, *p* = 0.019, $\eta_{p}^{2}=0.182$], again indicating that global and local adaptation showed different associations with the number of autistic traits of an individual.

These results show that with more autistic traits, there was a decrease in global adaptation, and a concomitant increase in local adaptation. These findings suggest that people with high levels of autistic traits indeed show decreased global processing (consistent with Experiment 1), but also use compensatory local processing to counteract the deficits in global processing. Notably, there was no significant difference in the sum of local and global adaptation strength between the two AQ groups [*t*(28) = 0.017, *p* = 0.97], indicating a compensatory mechanism adopted by observers with more autistic traits sufficed to reach the same level of discrimination performance as typical observers. However, this compensatory action fails in situations where only global processing can be applied, as is the case for location-invariant adaptation tested in the different-location condition of Experiment 2. As in Experiment 1, we also performed the analyses on just the female participants [in this experiment the mean AQ for males was 18.2 and for females 17.8; *t*(28) = 0.16, *p* = 0.88], and the results were robust to this change.

## Discussion

It has previously been suggested that people with ASD exhibit decreased high-level processing relative to low-level processing (Plaisted, 2001; Happé and Frith, 2006; Mottron et al., 2006), although unequivocal perceptual evidence has not been obtained (Dakin and Frith, 2005; Simmons et al., 2009). Here we employed biological motion stimuli and focused on the impact of autism traits on individual differences in biological motion processing. In two experiments we established that, within a population of typically developing healthy adults, people with more autistic traits show decreased global processing, and a compensatory increase in local processing. Specifically, we found that people with few autistic traits automatically process global aspects of biological motion even when this is detrimental to their central task performance. Such automatic processing of biological motion, which has been previously reported in the typical population (Thornton and Vuong, 2004), is indicative of a “hard-wired” bias toward global processing, because it cannot be switched off. Our study revealed individual differences on this automatic processing by showing that people with an increased number of autistic traits exhibit diminished or absent automatic processing of the global aspects of biological motion. If we extrapolate these data to the ASD population [which should be done with caution because the AQ score and ASD do not correlate perfectly (Baron-Cohen et al., 2001)], the absence of direct and involuntary processing of global information provides a possible explanation of why people with ASD show decreased social and contextual processing.

We also showed evidence for non-retinotopic adaptation effects (Kovacs et al., 2008). Moreover, we found that this global adaptation process is disrupted in people with many autistic traits, while, at the same time, local processing is enhanced in these observers. Interestingly, the decreased global processing observed in Experiment 2 did not manifest itself as a decrease in overall discrimination performance, but only in decreased adaptation strength, consistent with a previous study on face adaptation (Pellicano et al., 2007). Importantly, our study revealed that normal discrimination performance does not necessarily imply intact global processing, because people could rely more heavily on local processing to compensate for their impairment in global processing. When this compensation is successful, it can completely mask the presence of perceptual deficiencies at a particular level of processing in the visual hierarchy. This interpretation is consistent with recent functional magnetic resonance imaging (fMRI) research that has shown differences in brain activity between typically developing individuals and individuals with ASD, without finding behavioral differences (Kaiser and Pelphrey, 2012; McKay et al., 2012). The fact that our method can distinguish between such compensatory local processes and intact global processing is an important methodological advance.

Our data show that in the typically developing population, biological motion perception automatically depends on global information processing. However, global processing can be significantly weakened or even be absent due to observer characteristics such as an increased level of autistic traits, or due to stimulus characteristics such as scrambling, inverting or masking as used in many previous studies of biological motion perception. When global processing is made difficult or impossible, local processing may be recruited to partially counteract the loss of global processing. (This may be especially true in an adult population such as ours.) This interpretation reconciles apparently contradictory evidence that biological motion perception is generally a holistic process (e.g., Pavlova and Sokolov, 2000), and recent observations that local motion cues are sufficient (Troje and Westhoff, 2006), though not necessarily optimal (McKay et al., 2009), to perform certain biological motion discrimination tasks.

A central question in both vision science and autism research is how the interaction of local and global processing is orchestrated. Interpreting our data within a predictive coding framework provides insight into this question. A recently proposed Bayesian account offered by Pellicano and Burr (2012) could provide a similar answer, but lacks a clear link to possible neural mechanisms; van Boxtel and Lu (2013), see also Friston et al. (2013). Predictive coding schemes assume that the brain tries to predict the future, and discounts visual information that is consistent with the prediction (Rao and Ballard, 1999; Friston, 2010). Such discounting occurs in two stages: a high-level process makes predictions based on feedforward input from lower levels, and then feeds these predictions back to the lower level where they are subtracted from the input, potentially decreasing correlations, and redundancies between processing stages (Barlow and Foldiak, 1989), and also conserving energy (Baddeley, 1996). The better the prediction made at the higher stage, the lower the activity at lower levels. Predictive coding has been invoked to explain differences between tilt (i.e., low-level) and shape (i.e., higher-level) adaptation (He et al., 2012), and in the origin of V1 receptive field structure (Rao and Ballard, 1999). In our experiments, increased global processing could allow an observer to process biological motion stimuli as intact gestalts, which may improve predictions based on stored action templates. Thus, increased global processing leads to strengthened high-level adaptation, improved predictions, and lessened activation at lower levels in the visual system, and finally to weaker local adaptation. The predictive coding framework thus provides a powerful explanation of the experimental differences between people with low and high levels of autistic traits, and naturally suggests an inverse relationship between local and global processing by assuming that – consistent with our findings – people with high levels of autistic traits have decreased global processing of biological motion. It is of course possible that in addition to decreased global processing, increased or altered local processing (Mottron et al., 2006; Keita et al., 2011) contributes to the perceptual characteristics in people with many autistic traits. However, our experiments specifically controlled for local processing differences, and thus these differences are unlikely to have substantially contributed to our findings.

It is challenging to determine whether decreased global processing is due to a defect at the global stage, or due to diminished input. There is evidence that directing the observer’s attention to global information may decrease some of the high-level deficits in autism (Plaisted et al., 1999), suggesting that there may be no inherent problem with global processing (Mottron et al., 2006), but with a diminished input caused by decreased attention. Interestingly, in the predictive coding framework, attention is assumed to specifically influence feedforward connections, and thus inputs to the higher levels (Feldman and Friston, 2010), thereby potentially explaining how diminished global processing depends on attention. Decreased influences of global processing, and a malfunction of the predictive coding loop, may be due to decreased overall functional connectivity in the brain of people with ASD (Schipul et al., 2011). In fact, decreased functional connectivity between temporal and parietal areas, areas important in form and motion processing, respectively, has been reported in biological motion tasks in ASD (McKay et al., 2012).

Because of the natural way in which predictive coding explains why people with ASD are more focused on local cues than on global contextual cues, the predictive coding framework may be employed to account for other perceptual effects in autism. For example, the reported finding that people with autism are faster in finding embedded figures (Shah and Frith, 1983; Plaisted, 2001) is explained quite naturally through reduced predictive coding, because the reduced global processing reduces inhibition of the processing of the local embedded figure. Decreased predictive coding in people with autism predicts decreased contextual processing. (Experiments 1 and 2 may be construed as spatial and temporal examples of contextual influences, respectively.) Gestalt formation, as a prime example of contextual processing, should therefore be reduced in people with autism. Based on the predictive coding framework, we can make the more specific prediction that the reported reduction in brain activity in neurotypics in response to gestalts as compared to “broken” gestalts (Kubilius et al., 2011) should be absent in people with ASD. Finally, studies of the mirror-neuron system, which is essential to social understanding, have also been cast within a predictive coding framework (Kilner et al., 2007). Hence, the predictive coding framework potentially provides a parsimonious account for both perceptual and social problems in ASD.

## Conclusion

We investigated individual differences in the perception of biological motion. The present study revealed that people with high levels of autistic traits process biological motion differently than people with low levels of autistic traits. We found that an increased number of autistic traits correlates with decreased (automatic) global processing, and increased compensatory local processing. Compensatory local processing may make it also difficult to identify global deficits using some experimental paradigms. The present design, which holds stimuli constant across levels of visual processing, made it possible to clearly identify global deficits even in a subgroup of the typical population. Furthermore, we propose that a predictive coding framework not only explains our finding that people with more autistic traits have difficulty in global processing, and instead focus on local information, but also provides a general account of how certain perceptual and social deficits arise in ASD.

## Conflict of Interest Statement

The authors declare that the research was conducted in the absence of any commercial or financial relationships that could be construed as a potential conflict of interest.

## Footnotes

^1^A median split of the data, as done in Experiment 2 led to greatly unevenly sized groups in this experiment. So instead we decided to disregard the subjects with a median score. To make the groups of equal size we then took the nine remaining high-AQ subjects, and selected the nine subjects with the lowest AQ scores to compare them to. However, using the median split as done in Experiment 2 does yield nearly identical results. The only test that differed was the mixed-design repeated-measures ANOVA, which did not show a significant main effect of distractor anymore [*F*(2, 46) = 2.152, *p* = 0.128, $\eta_{p}^{2}=0.086$], but, importantly, it still showed a significant interaction [*F*(2, 46) = 3.4407, *p* = 0.041, $\eta_{p}^{2}=0.1301$].
